# Development of an *in vivo*, screenable, split-luciferase based model of huntingtin multimerization

**DOI:** 10.1016/j.isci.2026.116660

**Published:** 2026-07-07

**Authors:** Morgan G. Thomas, Simon A. Levy, Meredith H. Jenkins, Morgan Lambert, Bess Frost

**Affiliations:** 1 Center for Alzheimer’s Disease Research, Department of Molecular Biology, Cell Biology and Biochemistry, Brown University, Providence, RI, USA; 2 Department of Cell Systems and Anatomy, University of Texas Health San Antonio, San Antonio, TX, USA

**Keywords:** Huntington’s disease, huntingtin, polyglutamine, Drosophila, split-luciferase

## Abstract

Huntington’s disease is a neurodegenerative disorder caused by a polyglutamine (polyQ) expansion in exon one of the gene that encodes for the protein huntingtin (HTT). PolyQ expansion drives HTT aggregation into multimeric species that range from soluble oligomers to fibrillar, insoluble inclusion bodies. Cellular mechanisms facilitating HTT aggregation are incompletely understood, hindering efforts to develop strategies that prevent inclusion body formation or promote clearance of misfolded protein. To enable future unbiased *in vivo* screening approaches to identify genetic modifiers and pharmacological strategies to suppress HTT aggregation, we have developed HTT^LUM^, a split-luciferase-based detector of HTT-HTT interaction in adult *Drosophila melanogaster* neurons. This system permits real-time monitoring of HTT multimerization in living, active flies. The non-lethal nature of the HTT^LUM^ system enables subsequent analysis of HTT aggregation, neurotoxicity, and other phenotypes in the same flies, thus serving as a platform for medium-throughput screening followed by mechanistic validation of potential modifier candidates.

## Highlights

• HTT^LUM^ leverages split-luciferase to detect HTT multimerization in living flies
• HTT-induced neurotoxicity can be quantified by assessing brain vacuolization
• Proof-of-principle testing shows Fosfosal suppresses HTT Q93 aggregation
• HTT^LUM^ was developed for future genetic and small-molecule screening efforts

## Graphical abstract

**Figure:**
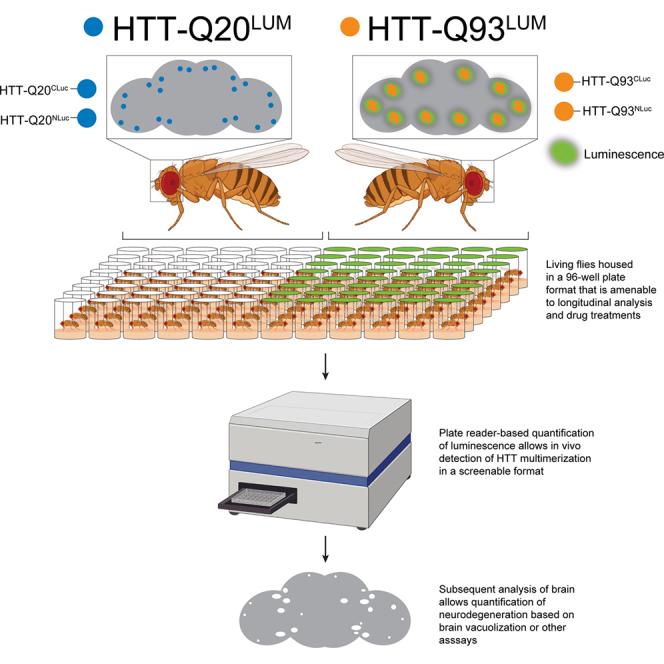

## Teaser

Neuroscience

## Introduction

Huntington’s disease is an autosomal dominant neurodegenerative disorder caused by an expanded CAG repeat (polyglutamine (polyQ)) in exon one of the huntingtin *(HTT)* gene.^1^ HTT is a large 348 kDa protein involved in a range of cellular functions, including gene transcription, RNA splicing, and intracellular transport.^1^^,^^2^ While HTT has fewer than 36 glutamines in most individuals, glutamine repeats greater than 40 cause Huntington’s disease in all cases; repeat lengths between 36 and 39 result in incomplete penetrance.^3^^,^^4^ Mutant HTT harboring expanded polyQ repeats aggregates to form a range of multimeric species, ranging from soluble oligomers to fibrillar inclusion bodies.^5^^,^^6^ While various multimeric species are detected in Huntington’s disease,^7^ recent evidence points toward oligomers as a likely causative agent of toxicity.^8^

While HTT multimerization involves the transition of its conformationally flexible polyQ stretch to a beta-sheet-rich structure,^9^^,^^10^^,^^11^^,^^12^ the cellular factors that facilitate the transition from monomers to oligomers and inclusion bodies remain to be elucidated.^13^ A strong propensity for aggregation is shared with several other proteins containing long stretches of glutamine residues; these neurodegenerative disorders are collectively referred to as “polyglutamine disorders.”^14^ While *in silico* modeling,^13^^,^^15^ *in vitro* biochemical studies,^6^^,^^15^^,^^16^^,^^17^^,^^18^ cultured cells,^5^^,^^18^^,^^19^^,^^20^ and vertebrate animal models of polyQ disorders^5^^,^^19^^,^^20^^,^^21^^,^^22^ have previously been utilized to gain some insight into the cellular mechanisms underlying the formation of polyQ inclusions, an *in vivo* platform to monitor HTT multimerization in a living, adult model system that is amenable to unbiased genetic or pharmacological screens is currently lacking in the field.

In the current study, we have developed HTT^LUM^, a *Drosophila* split-luciferase-based model that facilitates real-time quantification of HTT multimerization in living, active flies housed in individual wells of a 96-well plate. Split-luciferase assays facilitate quantitative measurement of protein-protein interaction. In this method, the luciferase enzyme is split into N- and C-terminal fragments, each of which is fused to a protein of interest. When proteins of interest interact, luciferase fragments are brought into close proximity and luminesce in the presence of the luciferase substrate D-luciferin.^23^^,^^24^ A key advantage of the HTT^LUM^ system is the capacity for medium-throughput luminescence-based screening of pharmacological agents or genetic modifiers that reduce HTT multimerization. In addition to luminescence-based assessment of HTT multimerization, we include two additional approaches that can be used to functionally validate candidate modifiers of HTT aggregation and demonstrate how this platform can be paired with subsequent assays to assess neurodegeneration in the same flies in which luminescence measurements were acquired. As a proof-of-principle, we demonstrate that the HTT^LUM^ system is sensitive to Fosfosal, a compound previously reported to suppress polyQ aggregation and neurotoxicity.^25^ Together, these features establish HTT^LUM^ as an *in vivo* pipeline for primary screening of a living, active model organism that can be followed by mechanistic validation of modifiers of HTT toxicity and multimerization.

## Results

### Creation of the HTT^LUM^ system for monitoring HTT multimerization in the adult *Drosophila* brain

**Figure 1:**
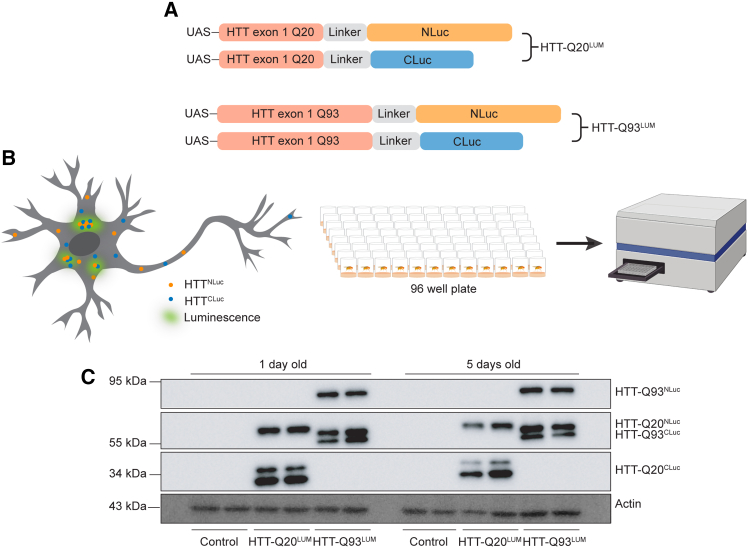
The HTT^LUM^ system (A) Gene models for transgenes used in the HTT^LUM^ system. (B) Schematic overview of the HTT^LUM^ model and conceptual cartoon of the bioluminescence measurement setup. (C) Western blot-based quantification of HTT exon 1 protein levels in HTT-Q20^LUM^ and HTT-Q93^LUM^*Drosophila* heads at day one and five of adulthood.

To engineer a new *Drosophila* model that enables split luciferase-based quantification of polyQ multimerization *in vivo*, we selected an expanded polyQ repeat length of 93 (Q93) for the disease model, and a polyQ repeat length of 20 (Q20) as a disease-relevant control for nonspecific effects of transgenic protein overexpression. We generated four constructs consisting of human *HTT* exon 1 harboring either Q20 or Q93 repeats, followed by a short, flexible glycine/serine linker that precedes either the N-terminus of click beetle luciferase (HTT-Q20^NLuc^, HTT-Q93^NLuc^) or the C-terminus of click beetle luciferase (HTT-Q20^CLuc^, HTT-Q93^CLuc^). Flexible linkers were included to ensure that N- and C- luciferase fragments have sufficient spatial freedom and flexibility to orient themselves and reconstitute a functional enzyme upon interaction of the target proteins. Constructs were introduced into pUA vectors, which adds ten 5′ upstream activating sequences (UASs) that facilitate tissue-specific *in vivo* transgene expression via the GAL4/UAS system (Figure 1A). Transgenes were inserted into specific sites in the *Drosophila* genome via embryo injection and PhiC31 site-specific integration. This system was designed such that N- and C-terminal luciferase fragments are brought into close proximity upon HTT exon 1 multimerization, which will promote partial reconstitution of enzymatic activity.^26^ After feeding *Drosophila* D-luciferin, the substrate of luciferase, HTT exon 1 multimerization can be quantified by measuring bioluminescence. To facilitate future screening efforts, individual flies can be housed in wells of a 96-well plate, and luminescence measurements can be collected via plate reader (Figure 1B).

Following oocyte injection and breeding of new *Drosophila* stocks, we next performed genetic recombination within Q20 models (HTT-Q20^NLuc^+HTT-Q20^CLuc^) and within Q93 models (HTT-Q93^NLuc^+HTT-Q93^CLuc^) to generate HTT-Q20^LUM^ and HTT-Q93^LUM^ models, both of which are on the third chromosome. We validated successful insertion of transgenes via DNA sequencing of adult flies; full sequences are provided in Table S1. Using the *elav-GAL4* driver to promote transgenic HTT exon 1 expression specifically in neurons, we find that all components of the HTT^LUM^ systems are effectively expressed in heads of HTT-Q20^LUM^ and HTT-Q93^LUM^ flies at days one and five of adulthood based on western blotting (Figures 1C and S1A). We observe a double band for the CLuc fusion proteins in both the HTT-Q20^LUM^ and HTT-Q93^LUM^ models. Treatment of protein lysates with phosphatase prior to blotting does not alter the abundance of the upper band within the doublet (Figure S1B), suggesting that it is not the result of post-translational phosphorylation. PCR-based amplification of the HTT-Q20^LUM^ and HTT-Q93^LUM^ transgenes reveals a single band (Figure S1C), suggesting that the doublet is not due to instability of polyQ repeats at the DNA level. While DNA sequence analysis does not support the existence of an alternative transcription start site, an additional ATG seven codons downstream of the initial HTT exon 1 ATG could serve as an alternative start site for translation, although this ATG is also present in NLuc-tagged constructs, which only have a single HTT protein band based on western blotting*.* Human HTT transcripts are approximately 30X greater than endogenous *Drosophila* huntingtin (Figure S1D) but do not differ between HTT-Q20^LUM^ and HTT-Q93^LUM^ models. HTT protein levels are not high enough to be detected by Ponceau S staining of western blotting membranes (Figure S1E).

### *In vivo* luminescence, *in situ* immunofluorescence, and protein solubility-based detection of HTT multimerization in HTT-Q93^LUM^*Drosophila*

**Figure 2:**
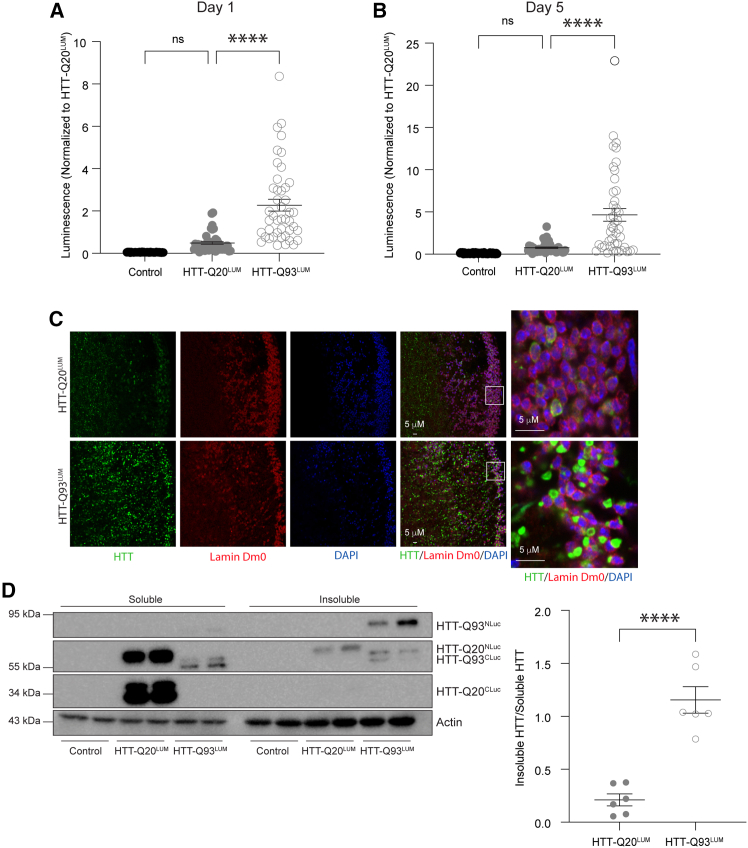
Luminescence, immunofluorescence, and protein solubility-based detection of HTT multimerization in the HTT^LUM^ system (A and B) Luminescence-based quantification of HTT multimerization in nontransgenic control, HTT-Q20^LUM^ and HTT-Q93^LUM^*Drosophila* at one (A) and five (B) days of age, *n* = 22 per group. Measurements are normalized to HTT-Q20^LUM^ within sex. (C) Immunofluorescence-based detection of HTT exon 1 and lamin Dm0 in HTT-Q20^LUM^ and HTT-Q93^LUM^*Drosophila* at day five of adulthood. Scale bars are 5 μm. (D) Western blot-based detection of soluble vs. insoluble HTT exon 1 protein in HTT-Q20^LUM^ and HTT-Q93^LUM^ at day five of adulthood, *n* = 6 per group. Data include an equal number of males and females per condition. ∗∗∗∗*p* < 0.0001, two-way ANOVA. Error bars reflect SEM.

Having established robust expression of all constituent transgenes in the HTT^LUM^ models, we next investigated whether the HTT^LUM^ system can be used to quantify HTT exon 1 multimerization *in vivo*. Flies were transferred to food containing D-Luciferin, the substrate for click beetle luciferase, for 24 h prior to transfer to single wells of a 96-well plate. We utilized *Drosophila* carrying only the *elav-GAL4* driver as an additional nontransgenic control. HTT-Q93^LUM^ flies exhibit increased luminescence at day one (Figures 2A and S2A) and five (Figures 2B and S2B) of adulthood compared to HTT-Q20^LUM^ and nontransgenic controls. As luminescence is significantly elevated in HTT-Q93^LUM^ at both one and five days of age, flies were aged to five days for all subsequent experiments.

To determine if HTT forms visible puncta suggestive of aggregation in the HTT-Q93^LUM^ model, we next performed immunofluorescence-based detection of human HTT in the adult *Drosophila* brain. Human HTT is undetectable in nontransgenic control flies, and its localization differs markedly between HTT-Q20^LUM^ vs. HTT-Q93^LUM^ flies (Figures 2C and S2C). While HTT exon 1 is primarily localized to the neuropil in the HTT-Q20^LUM^ model, HTT-Q93^LUM^ exhibits a strong HTT exon 1 localization to the soma. Large puncta are easily discernible in HTT-Q93^LUM^ *Drosophila*; co-immunofluorescence with an antibody detecting lamin Dm0, a protein that lines the internal surface of the nucleus, reveals HTT puncta adjacent to and within nuclei. HTT puncta range in size from 0.5–1.9 μm, in line with previous *Drosophila* models of HTT exon 1 polyQ expansion.^27^

As an additional assay to assess HTT aggregation and to determine if a portion of multimeric HTT species is insoluble, we next performed solubility fractionation to quantify the ratio of insoluble to soluble HTT in HTT-Q20^LUM^ and HTT-Q93^LUM^ models. We find that HTT-Q93^LUM^ has a significantly higher proportion of insoluble HTT protein compared to HTT-Q20^LUM^ (Figures 2D and S2D). Taken together, these analyses indicate that the extent of HTT exon 1 multimerization can be quickly assessed via non-lethal analysis in a 96-well plate, which can be followed by validation via immunofluorescence or solubility-based assays.

### Quantification of neurodegeneration in HTT^LUM^ models

**Figure 3:**
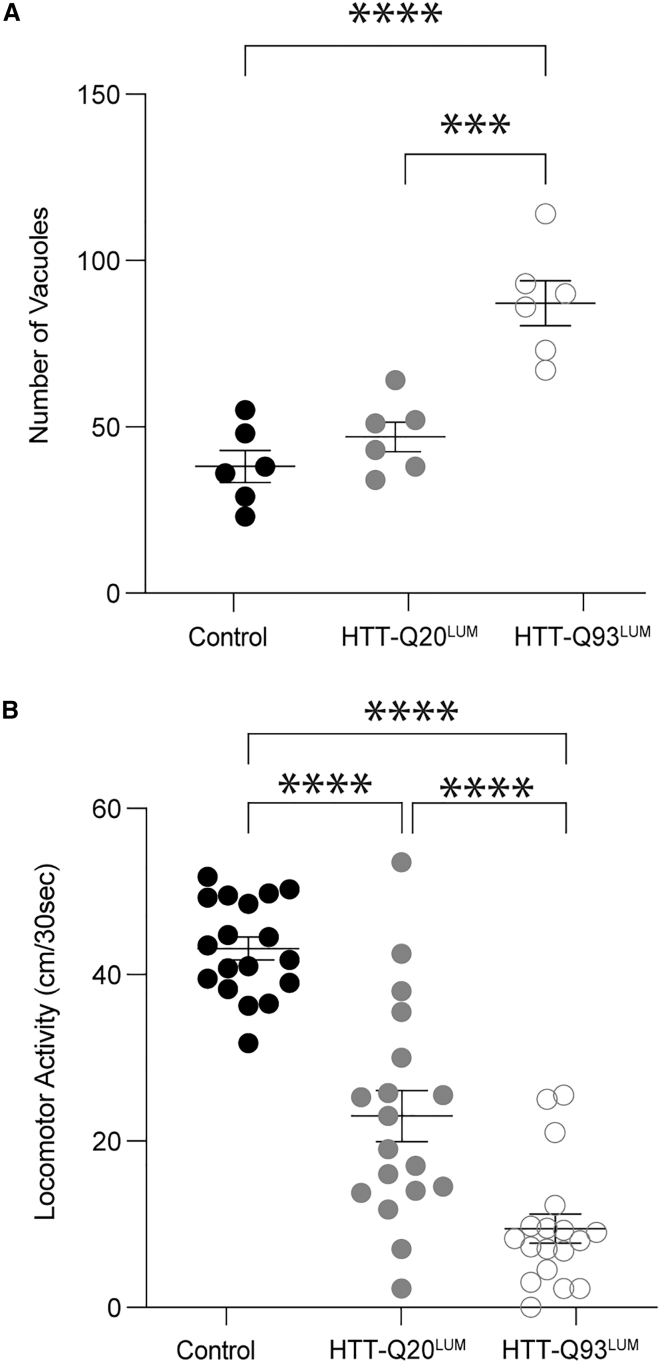
Quantification of neurotoxicity in HTT^LUM^ models (A and B) H&E staining-based quantification of brain vacuolization (A) and locomotor activity (B) in nontransgenic control, HTT-Q20^LUM^, and HTT-Q93^LUM^*Drosophila* at day five of adulthood. *n* = 6 for H&E, *n* = 18 for locomotor activity. Data include an equal number of males and females per condition. ∗∗∗*p* = 0.0003 and ∗∗∗∗*p* < 0.0001, one-way ANOVA. Error bars reflect SEM.

As luminescence-based quantification of HTT multimerization is non-lethal in the HTT^LUM^ models, neurotoxicity can be assessed in the same animals that were subject to luminescence measurements. To examine neurodegeneration in the HTT^LUM^ system, we first quantified the number of brain vacuoles in five-day-old HTT-Q20^LUM^ and HTT-Q93^LUM^ flies via hematoxylin and eosin staining (H&E). While HTT-Q20^LUM^ and nontransgenic control have equivalent levels of brain vacuolization, we find that HTT-Q93^LUM^ flies have significantly increased number of brain vacuoles (Figure 3A), consistent with neurodegeneration.

As Huntington’s disease is classified as a motor disorder due to the development of chorea and dystonia in afflicted individuals, we next assessed locomotor activity as an indicator of motor dysfunction in the HTT^LUM^ models. While HTT-Q93^LUM^ flies have a significant locomotor deficit compared to HTT-Q20^LUM^ and nontransgenic control flies, we find that HTT-Q20^LUM^ flies also have significantly reduced locomotor activity compared to nontransgenic control (Figure 3B).

### Fosfosal reduces HTT^LUM^ Q93 luminescence and neurotoxicity

**Figure 4:**
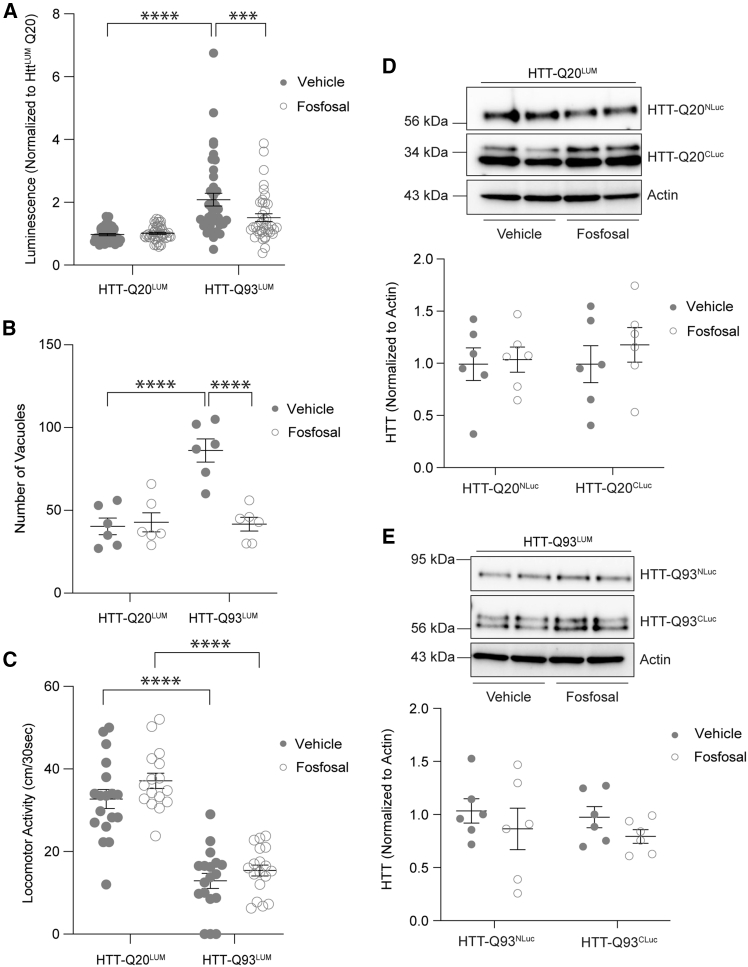
Fosfosal reduces HTT exon 1 Q93 multimerization and brain vacuolization (A–E) Luminescence (A), H&E-based quantification of brain vacuolization (B), and locomotor activity (C) in HTT-Q20^LUM^ and HTT-Q93^LUM^*Drosophila* at five days of age after 24 h treatment with 5 μM Fosfosal or vehicle (water). HTT protein levels are unaffected by Fosfosal treatment based on western blotting in HTT-Q20^LUM^ (D) or HTT-Q93^LUM^ (E) *Drosophila. n* = 22 per group for luminescence, *n* = 6 per group for H&E, *n* = 18 per group for locomotor activity, and *n* = 6 for western blotting. Data include an equal number of males and females per condition. ∗∗∗*p* < 0.001 and ∗∗∗∗*p* < 0. 0001 (two-way ANOVA). Error bars reflect SEM.

The HTT^LUM^ model was engineered as a platform for future *in vivo* screening efforts to identify pharmacologic suppressors of polyQ aggregation. As a proof-of-concept, we next determined if the HTT^LUM^ system is sensitive to a compound previously reported to suppress Q93 aggregation and toxicity. Fosfosal, a salicylic acid derivative with anti-inflammatory activity that is FDA-approved to treat inflammatory disorders, reduces HTT polyQ aggregation in cell culture; a previous dose response analysis suggests that 5 μM Fosfosal is sufficient to reduce polyQ-induced neurodegeneration in the *Drosophila* eye.^25^ After 24 h of exposure to 5 μM Fosfosal and D-Luciferin in food, we find that Fosfosal-treated HTT-Q93^LUM^ flies have significantly decreased luminescence compared to vehicle-treated HTT-Q93^LUM^ flies. Fosfosal has no effect on HTT-Q20^LUM^ controls (Figures 4A and S3A).

Having validated that Fosfosal reduces HTT exon 1 Q93 multimerization in the HTT-Q93^LUM^ model, we next determined whether Fosfosal reduces HTT-Q93^LUM^ neurotoxicity. While 24 h of 5 μM Fosfosal treatment significantly reduces brain vacuoles in the HTT-Q93^LUM^ model (Figure 4B), Fosfosal treatment does not significantly suppress HTT-induced locomotor deficits (Figure 4C), further suggesting that reduced locomotor activity may be a nonspecific consequence of transgene overexpression. Total levels of transgenic HTT are unaffected by Fosfosal treatment based on western blotting (Figures 4D, 4E, and S3B), indicating that reduced luminescence-based detection of HTT exon 1 Q93 multimerization is not a simple consequence of reduced transgene expression. Taken together, these data establish the HTT^LUM^ system as a robust model for *in vivo* detection of HTT-Q93 multimerization that is suitable for future screening efforts to identify compounds that suppress polyQ aggregation and neurodegeneration.

## Discussion

There is currently no cure for Huntington’s disease; available treatments only address symptoms and do not slow or halt disease progression. The overall goal of the current study was to develop a screenable system in which a large number of compounds could first be tested relatively quickly for their effects on polyQ multimerization in a living, awake model organism, followed by validation and mechanistic analyses on the effects of lead candidates on polyQ aggregation and neurotoxicity. HTT^LUM^ is a split luciferase-based *Drosophila* platform that includes HTT-Q93^LUM^ and HTT-Q20^LUM^. Both models are based on transgenic expression of human HTT exon 1 harboring either a pathogenic polyQ repeat length of 93 or a physiological polyQ repeat length of 20, which serves as a control for transgenic protein overexpression. While the modular GAL4/UAS system allows the user to drive HTT^LUM^ expression in any tissue/cell type of choice, the effects of pan-neuronal HTT^LUM^ expression are tested in the current study.

We observe a double band in HTT^LUM^ CLuc fusions that is unlikely to be due to an alternative transcription start site or post-translational phosphorylation. The double band is not present in NLuc fusions, nor was it observed in a similar tau-based model leveraging the same linker and CLuc sequence inserted into the same locus as the HTT^LUM^ CLuc models,^28^ suggesting that the doublet is not an artifact of the LUM system. While an ATG seven codons downstream of the original ATG within HTT exon 1 may provide an alternative start site for translation, this codon is also present in the NLuc models and would only result in the addition of approximately 1 kD. We thus speculate that the double banding pattern within HTT^LUM^ CLuc fusions is the result of a posttranslational modification such as SUMOylation, ubiquitination, O-GlcNAcylation, or acetylation.

We find that HTT-Q93^LUM^ exhibits higher levels of luminescence compared to HTT-Q20^LUM^. As future screening efforts will require subsequent validation with orthogonal approaches to ensure that lead compounds effectively reduce polyQ aggregation, we tested two additional approaches to quantify HTT-Q93 multimerization. We find that HTT forms punctate structures in the HTT-Q93^LUM^ model that can be easily discerned via immunofluorescence, and that HTT-Q93^LUM^ flies have significantly higher levels of insoluble HTT protein compared to HTT-Q20^LUM^ flies, suggestive of aggregation. HTT exon 1 puncta are present near and within nuclei in HTT-Q93^LUM^ *Drosophila*, consistent with prior observations in transgenic mouse models and postmortem human brains in which HTT polyQ aggregates appear adjacent to and within the nucleus; such aggregates may disrupt nuclear integrity and drive consequent cytotoxicity.^20^^,^^29^^,^^30^^,^^31^^,^^32^

We evaluated brain vacuolization and locomotor activity as two different measures of HTT-Q93-induced toxicity in the HTT^LUM^ system. While we find that brain vacuolization is increased in HTT-Q93^LUM^ *Drosophila* compared to HTT-Q20^LUM^ and nontransgenic *Drosophila* as expected*,* we were surprised to find decreased locomotor activity in HTT-Q20^LUM^ compared to nontransgenic *Drosophila*. As polyQ repeats less than 36 are generally not toxic in humans, and previous studies in *Drosophila* establish that transgenic expression of human HTT exon 1 harboring 25 polyQ repeats does not affect motor function, neurodegeneration, or lifespan,^33^ we speculate that the negative effects of HTT-Q20^LUM^ on *Drosophila* locomotor activity are a result of transgene overexpression rather than polyQ-induced neurotoxicity. *In vivo*, plate-reader-based luminescence detection of HTT-HTT interaction requires a degree of overexpression that likely exceeds that of previously developed *Drosophila* models; human HTT transcript levels are 30X higher than endogenous *Drosophila* huntingtin in the HTT^LUM^ models. While previous models did not quantify expression levels of transgenic vs. endogenous huntingtin, the VK27 and VK33 transgene insertion sites used in the current study are known to drive higher levels of transgene expression than the ZH-51D attP site used in previous work.^33^^,^^34^

We tested the effects of the salicylic acid derivative Fosfosal for its ability to suppress HTT polyQ aggregation and associated neurotoxicity as a proof-of-principle for future drug screening efforts. We find that a 24 h exposure to Fosfosal significantly reduces luminescence in HTT-Q93^LUM^ flies, indicating decreased HTT self-association. While these results suggest that Fosfosal interferes with multimerization, future studies utilizing solubility fractionation will be valuable to determine if a reduction in HTT-HTT interaction translates to reduced long-term accumulation of larger, SDS-insoluble aggregates. We find that Fosfosol treatment reduces brain vacuolization in HTT-Q93^LUM^ flies but does not suppress HTT-Q93^LUM^-induced locomotor deficits. While it is possible that a short treatment of Fosfosal is sufficient to reduce HTT-Q93 multimerization and neurotoxicity at a cellular level, while a longer treatment is required to attenuate physical symptoms that require network-level coordination or may be impacted by residual neuroinflammation, we speculate that nonspecific effects of protein overexpression on locomotor activity may also confound a potential positive effect of Fosfosol.

Overall, our studies establish HTT^LUM^ as a promising framework with which to interrogate polyQ multimerization and neurotoxicity. Luminescence can be acquired at single time points or longitudinally in live animals using a plate reader, allowing *in vivo* tracking of HTT polyQ multimerization in the context of aging and/or pharmacological intervention and subsequent use of brain tissue for analysis of neurotoxicity. Combined with an extensive genetic toolkit and ease of manipulating specific genes of interest in *Drosophila,* the HTT^LUM^ system is a powerful new model for identifying new cellular factors and compounds that mediate polyQ aggregation and toxicity.

### Limitations of the study

Brain vacuolization and locomotor activity were evaluated as two different methods to quantify neurotoxicity in HTT^LUM^ models. While HTT-Q93^LUM^ has significantly higher levels of brain vacuolization and significantly less locomotor activity compared to HTT-Q20^LUM^ and a nontransgenic control, we find that HTT-Q20^LUM^ also negatively affects locomotor activity compared to the nontransgenic control. Based on this finding, we suggest that future efforts utilize neurotoxicity assays that focus specifically on the brain, as the negative effects of HTT-Q20 ^LUM^ on locomotor activity may result from a nonspecific toxic effect of transgene overexpression.

## Resource availability

### Lead contact

Further information and request for resources and reagents should be directed to and will be fulfilled by the lead contact, Bess Frost (bess_frost@brown.edu).

### Materials availability

All unique reagents generated in this study, including all HTT^LUM^ transgenic lines, are available from the lead contact upon request.

### Data and code availability

• All raw and processed data generated in this paper are available from the lead contact upon request.
• This study does not report original code.
• Any additional information required to reanalyze the data reported in this study is available from the lead contact upon request.

## Acknowledgments

This study was supported in part by the National Institute of Neurological Disorders and Stroke T32 NS082145 (MGT). The *elav-GAL4* line was obtained from the Bloomington *Drosophila* Stock Center (NIH P40OD018537). *Drosophila* actin and lamin Dm0 antibodies (developed by Dr. Jim (Jung-Ching) Lim and Dr. Paul Fisher, respectively) were obtained from the Developmental Studies Hybridoma Bank, created by the NICHD of the NIH and maintained at The University of Iowa, Department of Biology, Iowa City, IA 52242. We would also like to acknowledge Dr. Yutaka Yamamoto for his expertise in *Drosophila* genetics.

## Author contributions

Conceptualization and formal analysis: B.F., S.A.L., and M.G.T.; investigation: M.G.T., S.A.L., M.J., and M.L.; writing and editing: M.G.T. and B.F.; figure preparation: M.G.T., S.A.L., and B.F. All authors read and approved the paper.

## Declaration of interests

The authors declare no competing interests.

## STAR★Methods

### Key resources table

**Table:** 

REAGENT or RESOURCE	SOURCE	IDENTIFIER
**Antibodies**		

Huntingtin	Invitrogen	PA5-85721; RRID: AB_2792860
Actin	Developmental Studies Hybridoma Bank	JLA20; RRID: AB_528068
Lamin Dm0	Developmental Studies Hybridoma Bank	ADL84; RRID: AB_528338
Anti-rabbit human ads-HRP	SouthernBiotech	4010-08; RRID: AB_2795919
Anti-mouse human ads-HRP	SouthernBiotech	1030-05; RRID: AB_2619742
Alexa Fluor 488 anti-rabbit	Invitrogen	A11008; RRID: AB_143165
Alexa Fluor 647 anti-mouse	Invitrogen	A21235; RRID: AB_2535804

**Bacterial and virus strains**		

pUA Plasmid	Han et al.^35^	Addgene 58372; RRID: Addgene_58372

**Chemicals, peptides, and recombinant proteins**		

DAPI Fluoromount-G	SouthernBiotech	0100–20
D-Luciferin	Thermo Scientific	PI88294

**Critical commercial assays**		

SuperSignal West Femto ECL kit	Thermo Scientific	34095
Lambda Protein Phosphatase	New England Biolabs	P0753S

**Experimental models: Organisms/strains**		

Elav-Gal4	Bloomington *Drosophila* Stock Center	458; RRID: BDSC_458
W1118	Bloomington *Drosophila* Stock Center	3605; RRID: BDSC_3605
Control: *elav-GAL4/+*	This paper	N/A
HTT-Q20^LUM^: *elav-GAL4/+;UAS-HTT-Q20-Nluc, UAS-HTT-Q20-CLuc/+*	This paper	N/A
HTT-Q93^LUM^: *elav-GAL4/+;UAS-HTT-Q93-Nluc, UAS-HTT-Q93-CLuc/+*	This paper	N/A

**Recombinant DNA**		

HTT-Q20-NLuc	This paper	N/A
HTT-Q20-CLuc	This paper	N/A
HTT-Q93-NLuc	This paper	N/A
HTT-Q93-CLuc	This paper	N/A

**Software and algorithms**		

GraphPad Prism	GraphPad Software	Prism10; RRID:SCR_002798
ImageJ	Schneider et al.^36^	https://imagej.net/ij/

**Other**		

*Drosophila* htt/FAM Forward Primer: CAACAGCTCGCTGGGAATTA Reverse Primer: CTTCGTTGACTCCGGCTC Probe Sequence: CAAGTTCCGTCGCTGATGAAGGG	Bio-Rad	N/A
Human HTT/FAM Forward Primer: CCTCAACCTCCTCCACAGG Reverse Primer: GAGGCTCCTCAGCCACA Probe Sequence: CCTCTGCTGCCTCAGCCACA	Bio-Rad	N/A
bGlu/HEX Forward Primer: CAGCGAGGACATGTGATTCT Reverse Primer: TTGCCGATCAGCATGGTATT Probe Sequence: CCAGTAGCTGCGGCTCGAAGA	Bio-Rad	N/A
HTT forward primer: CGACCCTGGAAAAGCTGATGA	IDT	N/A
NLuc Full Reverse: TTAGCCGTCGTCGTCGATG	IDT	N/A
CLuc Full Reverse: CTAACCGCCGGCCTTCTCC	IDT	N/A

### Experimental model and study participant details

#### Animal studies

Constructs for HTT-Q20^NLuc^, HTT-Q20^CLuc^, HTT-Q93^NLuc^, and HTT-Q93^CLuc^ were generated by GeneWiz and inserted into the pUA vector (Addgene #58372), which contains 10 UAS sequences.^35^ *Drosophila melanogaster* embryo injection of plasmids for PhiC31 integrase-mediated site-specific integration of transgenes was performed by BestGene Inc. NLuc-containing transgenes were inserted at site VK27 (integrase-carrying stock #9744); CLuc-containing transgenes were inserted at site VK33 (integrase-carrying stock #9759). Sequences of inserts are included in Table S1. HTT-Q20^NLuc^ and HTT-Q20^CLuc^ stocks were recombined to create HTT-Q20^LUM^; HTT-Q93^NLuc^ and HTT-Q93^CLuc^ stocks were recombined to create HTT-Q93^LUM^.

*Drosophila* were housed and crosses were performed at 25 °C with a 12 h light/dark cycle. Full genotypes are listed in the key resources table. Pan-neuronal expression of transgenes was achieved using the GAL4/UAS system with the *elav* promoter driving expression of the Gal4 transcription factor in all neurons of the *Drosophila* brain. *Elav-GAL4 Drosophila* were obtained from the Bloomington *Drosophila* Stock Center (stock #458). Flies were analyzed at days one and five of adulthood as noted in the main text and figure legends. An equal number of males and females were analyzed for all analyses; no significant sex differences were detected.

### Method details

#### Western blotting

One frozen *Drosophila* head per lane was homogenized in 15 μL 2x Laemmli buffer. After boiling for 10 min, samples were run on an 4–20% Tris-Glycine Mini Protein Gel (Invitrogen #XP04200BOX); equal loading of protein was assessed via Ponceau S staining after transfer to a nitrocellulose membrane. Membranes were washed in PBS+0.1% Tween 20 (PBS-Tw) and blocked for 30 min at room temperature in blocking buffer (2% w/v milk+PBS-Tw). Membranes were then incubated with primary antibody diluted in blocking buffer overnight at 4 °C on a rocker, washed in PBS-Tw, and incubated with an HRP-conjugated secondary antibody diluted in blocking buffer for 2 h at room temperature. Blots were developed using the SuperSignal West Femto ECL kit (ThermoFisher #34095); band intensities were quantified using ImageJ.^36^

#### Luminescence measurements

*Drosophila* were placed on food containing 15 mM D-Luciferin (Thermo Scientific #PI88294) for 24 h. On the day of measurement, flies were anesthetized and transferred to 96-well plates. The plate was then covered with a clear plastic adhesive cover (Sigma-Aldrich #Z721417) and transferred to the plate reader (GloMax Discover Microplate Reader Promega #GM3000). After allowing flies to recover from anesthesia for 30 min, luminescence signal from each well was collected for 10 s by a broad-spectrum luminescence reading with a wavelength range of 350–700 nm. HTT-Q93^LUM^ was normalized to HTT-Q20^LUM^ within sex. The average luminescence signal of male and female HTT-Q20^LUM^ flies was first calculated separately, after which all males were divided by the male average and all females were divided by the female average for each genotype or condition.

#### Immunofluorescence and histology

Adult *Drosophila* brains were dissected in PBS and fixed for 20 min in methanol. After washing with PBS +0.3% Triton X-100 (PBS-Tr), brains were incubated with blocking buffer (2% w/v milk in PBS-Tr) for 30 min, followed by overnight incubation with primary antibodies in blocking buffer at room temperature. After washing with 0.3% PBS-Tr, brains were incubated with Alexa Fluor-conjugated secondary antibodies in blocking buffer for 2 h at room temperature. Brains were washed with PBS-Tr and mounted in DAPI Fluoromount-G (SouthernBiotech #0100-20) to visualize nuclei. Images were acquired by confocal microscopy (Zeiss LSM 880) and analyzed using ImageJ.

H&E staining was performed on 4 μm sections of formalin fixed, paraffin embedded *Drosophila* brain tissue. Slides were deparaffinized in xylene 1 for 10 min, then quickly dipped in xylene 2. Slides were then quickly dipped in a series of 100% ethanol, then rinsed twice with water. Slides were then stained with Harris Modified Hematoxylin (Fisher Chemical #SH26-500D) for 1 min, rinsed three times with tap water and quickly dipped in acid alcohol (1% HCI, 70% ethanol). Slides were then rinsed three times with tap water and quickly dipped in 10% Eosin Y (Fisher Chemical #E511-100) in ethanol, and rinsed twice in 100% ethanol. Slides were then dehydrated through quick dips in ethanols and xylenes and mounted with Permount (Fisher Chemical #SP15-100). Brain vacuoles were counted throughout the entire brain using brightfield microscopy.

#### Protein solubility assay

Five frozen *Drosophila* heads per lane were homogenized in 35 μL of ice-cold Triton-X lysis buffer (1% Triton X-100, 50 mM Tris-HCl, 150 mM NaCl, 1 mM EDTA, and 1X Halt protease inhibitor cocktail). Samples were incubated on ice for 15 min then centrifuged at 16,000 g at 4 °C for 20 min. The supernatant was transferred to a new tube as the soluble fraction. The pellet was washed with cold Triton-X lysis buffer, then 25 μL of SDS solubilization buffer was added (50 mM Tris-HCl, 150 mM NaCl, 2% SDS). Samples were vortexed vigorously and incubated at 70 °C for 10 min. The supernatant was transferred to a new tube as the insoluble fraction. Both fractions were then mixed with Laemmli buffer and boiled for 10 min. Samples were then run on 4–20% Tris-Glycine Mini Protein Gel (Invitrogen #XP04200BOX), transferred and probed following the Western blot protocol outlined above.

#### RNA extraction, cDNA synthesis and digital droplet PCR (ddPCR)

Five frozen *Drosophila* heads were homogenized in 50 μL TRIzol (#15596026, ThermoFisher Scientific) with disposable pestle, then 450 μL of fresh TRIzol was added. Samples were inverted for 1 min and then centrifuged for 10 min at 12,000 x g at 4 °C. The supernatant was transferred to a new tube and incubated at room temperature for 5 min 100 μL of chloroform was added and samples were inverted for 1 min, incubated at room temperature for 3 min and then centrifuged for 15 min at 12,000 x g, 4 °C. The clear upper phase was removed and added to a new Eppendorf tube with 1:1 isopropanol. Samples were inverted, incubated at room temperature for 10 min, then centrifuged for 10 min at 12,000 x g, 4 °C. 500 μL of 75% ethanol was added to pellets, then samples were centrifuged for 5 min at 4 °C; this step was repeated four times. Pellets were air dried after the last wash, then resuspended in 10 μL of nuclease-free water. RNA concentration was measured using a NanoDrop 8000 spectrophotometer (ThermoFisher Scientific). 500 ng RNA was reverse transcribed to cDNA using the High-Capacity cDNA Reverse Transcription Kit (#4368814, ThermoFisher Scientific). Equal quantities of cDNA were loaded into QX200 Droplet PCR System (Bio-Rad). Probes for endogenous *Drosophila* huntingtin (FAM), human HTT (FAM), and β glucuronidase (βGlu, endogenous control, HEX) were custom-designed and purchased through Bio-Rad. *Drosophila* huntingtin and human HTT were each individually multiplexed with βGlu for normalization. Primer sequences are included in the key resources table.

#### Non-lethal PCR genotyping of adult *Drosophila*

Adult *Drosophila* were anesthetized, and both wings were removed with a razor blade distal to the wing base. Wing tissue was carefully placed at the bottom of a 0.2 mL PCR tube and covered with 10 μL of 400 μg/mL proteinase K in Buffer A (10 mM Tris-Cl, 1 mM EDTA, and 25 mM NaCl), ensuring that the wings were fully submerged. Samples were incubated at 37 °C for 1 h, then proteinase K was inactivated by heating at 95 °C for 2 min. The gene of interest was amplified using the Q5 PCR Kit (New England Biolabs, E0555S). For each reaction, 24 μL of master mix was added to a new PCR tube with 1 μL of DNA template. Tubes were briefly spun down and placed in a thermocycler. PCR products were sent to GeneWiz for sanger sequencing, primer sequences are included in the key resources table.

#### Protein phosphatase treatment

One frozen *Drosophila* head per lane was homogenized in 40 μL of ice-cold Triton-X lysis buffer (1% Triton X-100, 50 mM Tris-HCl, 150 mM NaCl, 1 mM EDTA, and 1X Halt protease inhibitor cocktail). Samples were incubated on ice for 5 min then briefly spun down to settle debris. The supernatant was transferred to a new tube with 10 μL of *λ*PP buffer (250 mM Tris-HCl pH 7.5 and 25 mM DTT), 1 μL 50 mM MnCl_2_, and 1 μL of H_2_O or 2 μL of NEB *λ*PP. Samples were incubated at 30 °C for 30 min. Samples were then subject to SDS-PAGE and Western blotting as described above.

#### Locomotor activity

*Drosophila* were singly housed in fresh vials on day four of adulthood; the assay was performed in the afternoon of day five. The number of centimeters walked in 30-s intervals was counted in four trials per fly, and an average of the trials was plotted.

#### Drug treatment

Solid fly food was melted by raising the temperature to 100 °C and stirred constantly until cooled to 60 °C. A 5 mg/mL stock solution of Fosfosal was diluted into 60 °C food to a final concentration of 5 μg/mL.

### Quantification and statistical analyses

An ordinary one-way ANOVA with Dunnett’s post-hoc test or two-way ANOVA with Sidak’s post hoc test were used to compare multiple means as appropriate. The experimental unit for all experiments was a single fly except for solubility assays, which represent five fly heads per lane. Data are presented as the mean ± SEM to reflect the precision of the mean estimates and to facilitate the comparison of experimental groups. *p* values and statistical tests are defined in the figure legends; ∗*p* < 0.05, ∗∗*p* < 0.01, ∗∗∗*p* < 0.001, ∗∗∗∗*p* < 0.0001.
