# Supplementary material for: Development of an in vivo, screenable, split-luciferase based model of huntingtin multimerization
Source: iScience. 2026 Jul 7;29(7):116660. doi: 10.1016/j.isci.2026.116660 (PMC13355796; doi:10.1016/j.isci.2026.116660)
Supplement: Document S1. Figures S1–S3 and Table S1 [file mmc1.pdf]

iScience, Volume *29*

Supplemental information

Development of an *in vivo*, screenable, split-luciferase based model of huntingtin multimerization

Morgan G. Thomas, Simon A. Levy, Meredith H. Jenkins, Morgan Lambert, and Bess Frost

SUPPLEMENTAL INFORMATION

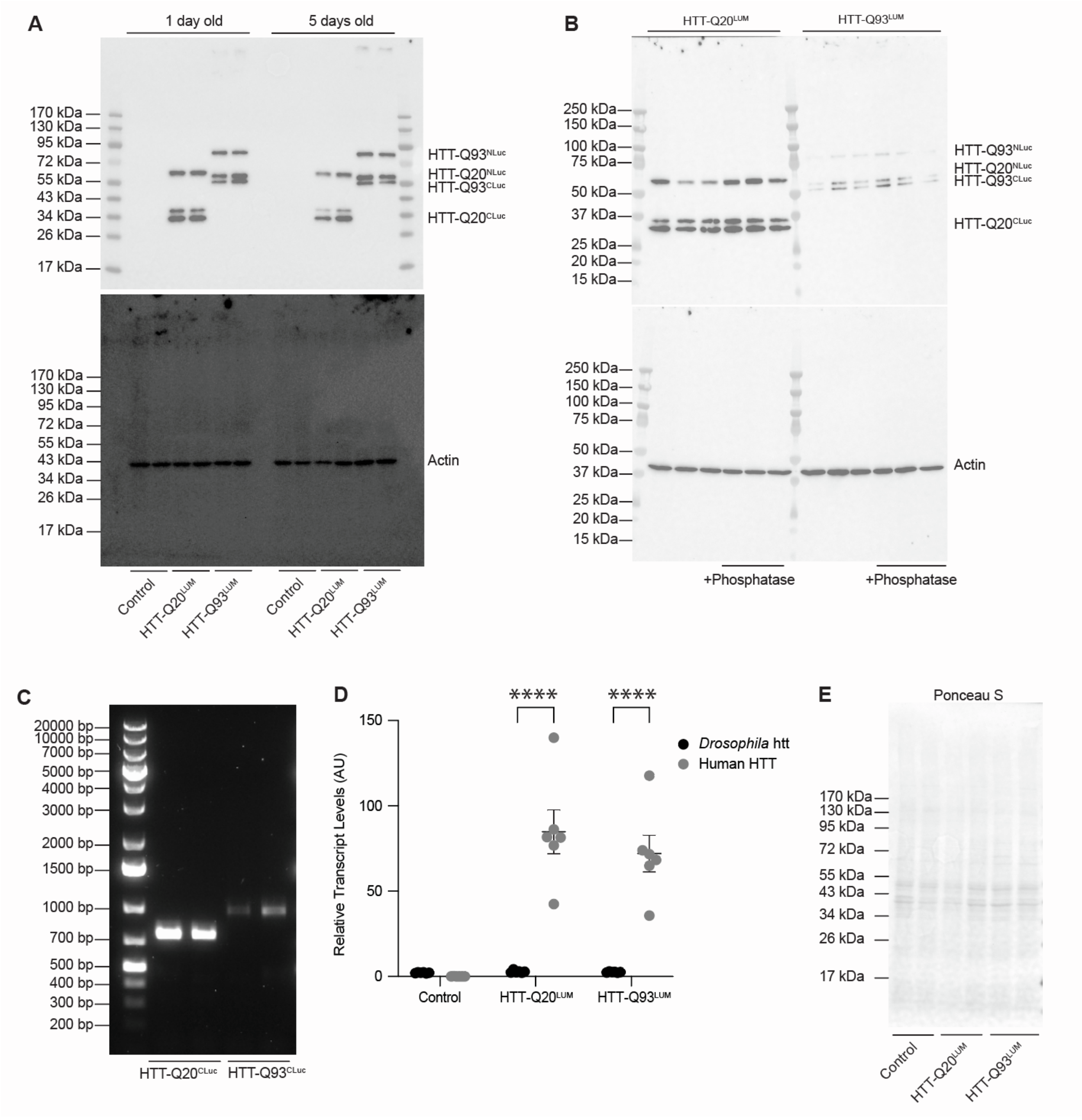

Figure S1: HTT protein, DNA and transcript levels in HTT^{LUM} models. A) Full Western blots of Fig. 1C. **B)** Phosphatase treatment of head protein lysates does not affect double banding pattern of HTT CLuc fusions. **C)** PCR-based amplification of CLuc HTT fusions only show a single DNA band based on agarose gel electrophoresis. **D)** ddPCR-based analysis of *Drosophila* htt and human HTT transcripts relative to βGlu, n=6, ****p<0.0001, ANOVA. **E)** Ponceau S staining of Western blotting membrane shows no prominent bands at the sizes of transgenic human HTT.

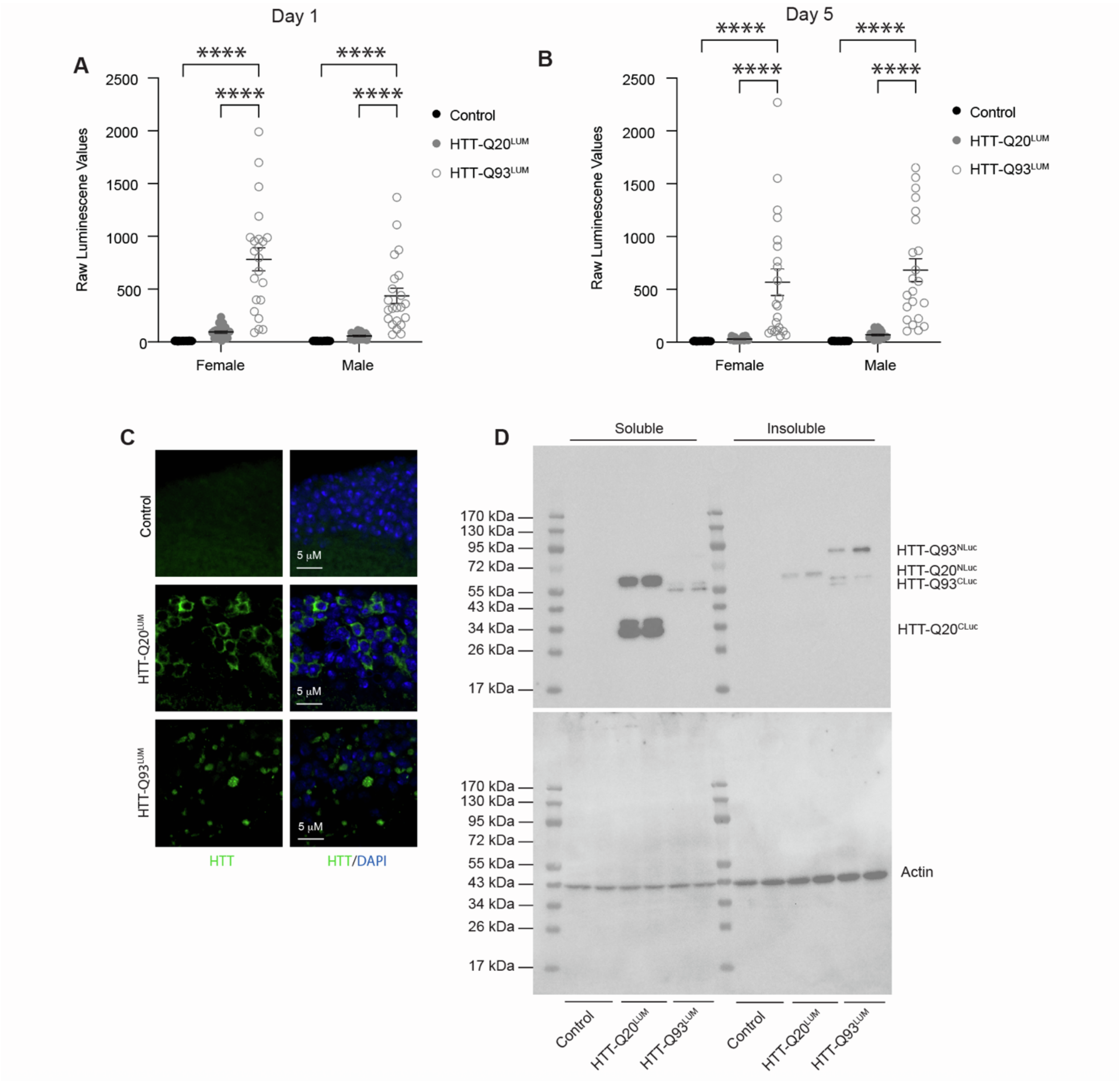

Figure S2: Raw luminescence measurements, HTT immunofluorescence and full Western blots of protein solubility assay. A, B) Raw luminescence measurements of HTT-Q20^{LUM} and HTT-Q93^{LUM} *Drosophila* from Fig. 2A. ****p<0.0001, two-way ANOVA. Error bars reflect SEM. **C)** Immunofluorescence-based detection of transgenic human HTT reveals no signal in the nontransgenic control. Scale bar is 5 µm. **D)** Full Western blots from Fig. 2D.

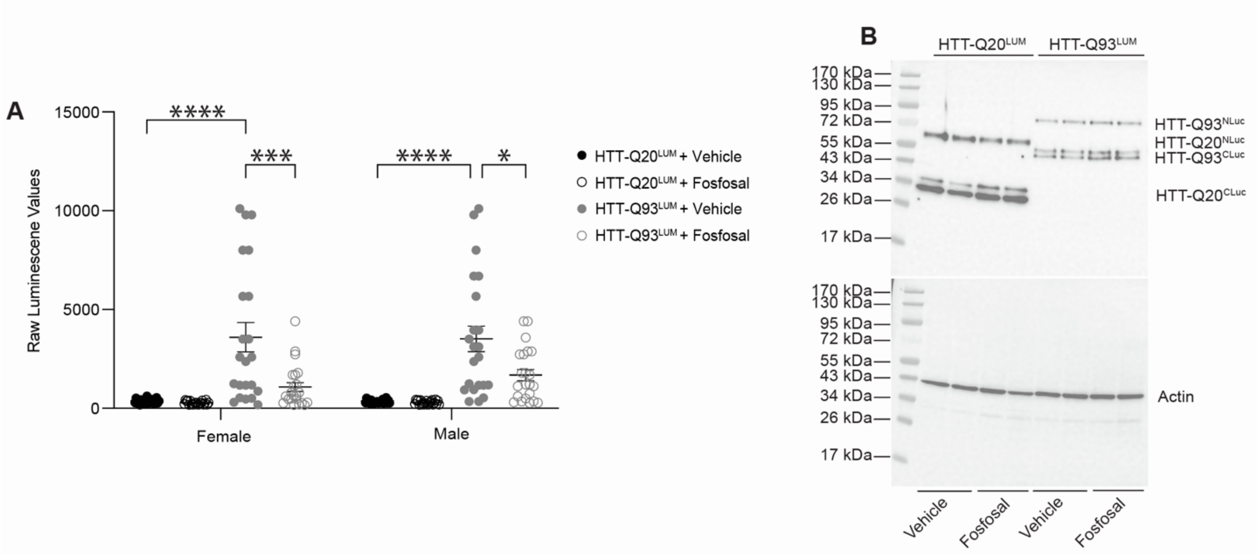

Figure S3: Raw luminescence measurements and full Western blots from Fosfosal treatments. A) Raw luminescence measurements from Fig. 4A. **B)** Full Western blots of Fig. D, E. *p<0.05, ***p<0.001 ****p<0.0001, two-way ANOVA. Error bars reflect SEM.

Kozak sequence, **Start codon**, HTT exon 1, Linker, CLuc/NLuc, **Stop codon**

HTT-Q93^{NLuc} complete sequence

*CAAA***ATG**GCGACCCTGGAAAAGCTGATGAAGGCCTTCGAGTCCCTCAAAAGCTTCCAACAGCAGCAACAGCAACAACAGCAGCAACAGC
AACAACAGCAGCAACAGCAACAACAGCAGCAACAGCAGCAACAGCAACAACAGCAGCAACAGCAACAACAGCAGCAACAGCAACAACA
GCAGCAACAGCAACAACAACAGCAGCAACAGCAACAACAGCAGCAACAGCAACAACAGCAGCAACAGCAACAACAGCAGCAACAGCAG
CAACAGCAACAACAGCAGCAACAGCAACAACAGCAGCAACAGCAACAACAGCAGCAACAGCAACAACAGCCGCCACCACCTCCCCCTCC
ACCCCCACCTCCTCAACTTCCTCAACCTCCTCCACAGGCACAGCCTCTGCTGCCTCAGCCACAACCTCCTCCACCTCCACCTCCACCTCCTCCA
GGCCCAGCTGTGGCTGAGGAGCCTCTGCACCGACCTGGCGGTGGCTCATCTGGCGGAGGTGTGAAGCGTGAGAAAAATGTCATCTATGG
CCCTGAGCCTCTCCATCCTTTGGAGGATTTGACTGCCGGCGAAATGCTGTTTCGTGCTCTCCGCAAGCACTCTCATTTGCCTCAAGCCTTGG
TCGATGTGGTCGGCGATGAATCTTTGAGCTACAAGGAGTTTTTTGAGGCAACCGTCTTGCTGGCTCAGTCCCTCCACAATTGTGGCTACAA
GATGAACGACGTCGTTAGTATCTGTGCTGAAAACAATACCCGTTTCTTCATTCCAGTCATCGCCGCATGGTATATCGGTATGATCGTGGCT
CCAGTCAACGAGAGCTACATTCCCGACGAACTGTGTAAAGTCATGGGTATCTCTAAGCCACAGATTGTCTTCACCACTAAGAATATTCTGA
ACAAAGTCCTGGAAGTCCAAAGCCGCACCAACTTTATTAAGCGTATCATCATCTTGGACACTGTGGAGAATATTCACGGTTGCGAATCTTT
GCCTAATTTCATCTCTCGCTATTCAGACGGCAACATCGCAAACTTTAAACCACTCCACTTCGACCCTGTGGAACAAGTTGCAGCCATTCTGT
GTAGCAGCGGTACTACTGGACTCCCAAAGGGAGTCATGCAGACCCATCAAAACATTTGCGTGCGTCTGATCCATGCTCTCGATCCACGCG
TGGGCACTCAGCTGATTCCTGGTGTCACCGTCTTGGTCTACTTGCCTTTCTTCCATGCTTTCGGCTTTAGCATTACTTTGGGTTACTTTATGG
TCGGTCTCCGCGTGATTATGTTCCGCCGTTTTGATCAGGAGGCTTTCTTGAAAGCCATCCAAGATTATGAAGTCCGCAGTGTCATCAACGT
GCCTAGCGTGATCCTGTTTTTGTCTAAGAGCCCACTCGTGGACAAGTACGACTTGTCTTCACTGCGTGAATTGTGTTGCGGTGCCGCTCCA
CTGGCTAAGGAGGTCGCTGAAGTGGCCGCCAAACGCTTGAATCTTCCAGGGATTCGTTGTGGCTTCGGCCTCACCGAATCTACCAGCGCT
AACATTCACTCTCTCGGGGATGAGTTTAAGAGCGGCTCTTTGGGCCGTGTCACTCCACTCATGGCTGCTAAGATCGCTGATCGCGAAACTG
GTAAGGCTTTGGGCCCGAACCAAGTGGGCGAGCTGTGTATCAAAGGCCCTATGGTGAGCAAGGGTTATGTCAATAACGTTGAAGCTACC
AAGGAGGCCATCGACGACGACGGC**TAA**

HTT-Q93^{CLuc} complete sequence

*CAAA***ATG**GCGACCCTGGAAAAGCTGATGAAGGCCTTCGAGTCCCTCAAAAGCTTCCAACAGCAGCAACAGCAACAACAGCAGCAACAGC
AACAACAGCAGCAACAGCAACAACAGCAGCAACAGCAGCAACAGCAACAACAGCAGCAACAGCAACAACAGCAGCAACAGCAACAACA
GCAGCAACAGCAACAACAACAGCAGCAACAGCAACAACAGCAGCAACAGCAACAACAGCAGCAACAGCAACAACAGCAGCAACAGCAG
CAACAGCAACAACAGCAGCAACAGCAACAACAGCAGCAACAGCAACAACAGCAGCAACAGCAACAACAGCCGCCACCACCTCCCCCTCC
ACCCCCACCTCCTCAACTTCCTCAACCTCCTCCACAGGCACAGCCTCTGCTGCCTCAGCCACAACCTCCTCCACCTCCACCTCCACCTCCTCCA
GGCCCAGCTGTGGCTGAGGAGCCTCTGCACCGACCTGGATCCGGCGGTGGCTCATCTGGCGGAGGTTCCGGTAAGGGTTATGTCAATAA
CGTTGAAGCTACCAAGGAGGCCATCGACGACGACGGCTGGTTGCATTCTGGTGATTTTGGATATTACGACGAAGATGAGCATTTTTACGT
CGTGGATCGTTACAAGGAGCTGATCAAATACAAGGGTAGCCAGGTTGCTCCAGCTGAGTTGGAGGAGATTCTGTTGAAAAATCCATGCAT
TCGCGATGTCGCTGTGGTCGGCATTCCTGATCTGGAGGCCGGCGAACTGCCTTCTGCTTTCGTTGTCAAGCAGCCTGGTAAAGAAATTACC
GCCAAAGAAGTGTATGATTACCTGGCTGAACGTGTGAGCCATACTAAGTACTTGCGTGGCGGCGTGCGTTTTGTTGACTCCATCCCTCGTA
ACGTAACAGGCAAAATTACCCGCAAGGAGCTGTTGAAACAATTGTTGGAGAAGGCCGGCGGT**TAG**

HTT-Q20^{NLuc} complete sequence

*CAAA***ATG**GCGACCCTGGAAAAGCTGATGAAGGCCTTCGAGTCCCTCAAAAGCTTCCAACAGCAGCAACAGCAACAACAGCAGCAACAGC
AACAACAGCAGCAACAACAGCAACAACCGCCACCACCTCCCCCTCCACCCCCACCTCCTCAACTTCCTCAACCTCCTCCACAGGCACAGCCT
CTGCTGCCTCAGCCACAACCTCCTCCACCTCCACCTCCACCTCCTCCAGGCCCAGCTGTGGCTGAGGAGCCTCTGCACCGACCTGGCGGTG

GCTCATCTGGCGGAGGTGTGAAGCGTGAGAAAAATGTCATCTATGGCCCTGAGCCTCTCCATCCTTTGGAGGATTTGACTGCCGGCGAAA<br>TGCTGTTTCGTGCTCTCCGCAAGCACTCTCATTTGCCTCAAGCCTTGGTCGATGTGGTCGGCGATGAATCTTTGAGCTACAAGGAGTTTTTT<br>GAGGCAACCGTCTTGCTGGCTCAGTCCCTCCACAATTGTGGCTACAAGATGAACGACGTCGTTAGTATCTGTGCTGAAAACAATACCCGTT<br>TCTTCATTCCAGTCATCGCCGCATGGTATATCGGTATGATCGTGGCTCCAGTCAACGAGAGCTACATTCCCGACGAACTGTGTAAAGTCAT<br>GGGTATCTCTAAGCCACAGATTGTCTTCACCACTAAGAATATTCTGAACAAAGTCCTGGAAGTCCAAAGCCGCACCAACTTTATTAAGCGT<br>ATCATCATCTTGGACACTGTGGAGAATATTCACGGTTGCGAATCTTTGCCTAATTTCATCTCTCGCTATTCAGACGGCAACATCGCAAACTT<br>TAAACCACTCCACTTCGACCCTGTGGAACAAGTTGCAGCCATTCTGTGTAGCAGCGGTACTACTGGACTCCCAAAGGGAGTCATGCAGAC<br>CCATCAAAACATTTGCGTGCGTCTGATCCATGCTCTCGATCCACGCGTGGGCACTCAGCTGATTCCTGGTGTCACCGTCTTGGTCTACTTGC<br>CTTTCTTCCATGCTTTCGGCTTTAGCATTACTTTGGGTTACTTTATGGTCGGTCTCCGCGTGATTATGTTCCGCCGTTTTGATCAGGAGGCTT<br>TCTTGAAAGCCATCCAAGATTATGAAGTCCGCAGTGTCATCAACGTGCCTAGCGTGATCCTGTTTTTGTCTAAGAGCCCACTCGTGGACAA<br>GTACGACTTGTCTTCACTGCGTGAATTGTGTTGCGGTGCCGCTCCACTGGCTAAGGAGGTCGCTGAAGTGGCCGCCAAACGCTTGAATCTT<br>CCAGGGATTCGTTGTGGCTTCGGCCTCACCGAATCTACCAGCGCTAACATTCACTCTCTCGGGGATGAGTTTAAGAGCGGCTCTTTGGGCC<br>GTGTCACTCCACTCATGGCTGCTAAGATCGCTGATCGCGAAACTGGTAAGGCTTTGGGCCCGAACCAAGTGGGCGAGCTGTGTATCAAAG<br>GCCCTATGGTGAGCAAGGGTTATGTCAATAACGTTGAAGCTACCAAGGAGGCCATCGACGACGACGGC**TAA**
**HTT-Q20^{CLuc} complete sequence**
*CAAA***ATG**GCGACCCTGGAAAAGCTGATGAAGGCCTTCGAGTCCCTCAAAAGCTTCCAACAGCAGCAACAGCAACAACAGCAGCAACAGC<br>AACAACAGCAGCAACAACAGCAACAACCGCCACCACCTCCCCCTCCACCCCCACCTCCTCAACTTCCTCAACCTCCTCCACAGGCACAGCCT<br>CTGCTGCCTCAGCCACAACCTCCTCCACCTCCACCTCCACCTCCTCCAGGCCCAGCTGTGGCTGAGGAGCCTCTGCACCGACCTGGATCCG<br>GCGGTGGCTCATCTGGCGGAGGTTCCGGTAAGGGTTATGTCAATAACGTTGAAGCTACCAAGGAGGCCATCGACGACGACGGCTGGTTG<br>CATTCTGGTGATTTTGGATATTACGACGAAGATGAGCATTTTTACGTCGTGGATCGTTACAAGGAGCTGATCAAATACAAGGGTAGCCAG<br>GTTGCTCCAGCTGAGTTGGAGGAGATTCTGTTGAAAAATCCATGCATTCGCGATGTCGCTGTGGTCGGCATTCCTGATCTGGAGGCCGGC<br>GAACTGCCTTCTGCTTTCGTTGTCAAGCAGCCTGGTAAAGAAATTACCGCCAAAGAAGTGTATGATTACCTGGCTGAACGTGTGAGCCATA<br>CTAAGTACTTGCGTGGCGGCGTGCGTTTTGTTGACTCCATCCCTCGTAACGTAACAGGCAAAATTACCCGCAAGGAGCTGTTGAAACAATT<br>GTTGGAGAAGGCCGGCGGT**TAG**

Table S1: DNA sequencing of *HTT* transgenes from DNA isolated from HTT-Q20^{LUM} and HTT-Q93^{LUM} *Drosophila*
